# Sheep in vivo models for evaluation of safety, functionality and regenerative potential of arterial grafts

**DOI:** 10.1016/j.mex.2026.104055

**Published:** 2026-07-19

**Authors:** Klas Österberg, Åsa Lindgren, Vijay Kumar Kuna, Nidal Ghosheh, Jane Synnergren, Patrik Stenlund, Yalda Bogestål, Sarunas Petronis, Simon Standoft, Veronica Crisostomo, Francisco M. Sanchez-Margallo, Claudia Baez-Diaz, Hardis Rabe, Joakim Håkansson

**Affiliations:** a Sahlgrenska Academy, Institution of Medicine, Department of Molecular and Clinical Medicine, Blå Stråket 5 B Wallenberg Laboratory, Gothenburg, 41345; b RISE Research Institutes of Sweden, Division of Material and Production, Department of Medical Device and Diagnostics, Brinellgatan 4, Borås, 504 62, Sweden; c Systems Biology Research Center, School of Bioscience, University of Skövde, Skövde, Sweden; d Department of Molecular and Clinical Medicine, Institute of Medicine, Sahlgrenska Academy, University of Gothenburg, Gothenburg, Sweden; e Jesús Usón Minimally Invasive Surgery Centre, Cáceres, 10004, Spain; f RICORS-TERAV Network, ISCIII, Madrid, 28029, Spain; g Institute of Biomedicine, Department of Infectious Diseases, The Sahlgrenska Academy, University of Gothenburg, Gothenburg, Sweden; h Department of Laboratory Medicine, Institute of Biomedicine, Sahlgrenska Academy at University of Gothenburg, Gothenburg, Sweden

**Keywords:** Vascular grafts, Advanced therapy medicinal product (ATMP), Arteries, Safety and functional evaluation, Large animal model, Method

## Abstract

Cardiovascular disease remains the leading cause of mortality worldwide. Although minimally invasive interventions have improved outcomes, open surgical reconstruction using vascular grafts is still required for many patients. Autologous vessels are preferred due to superior elastic modulus and biocompatibility; however, a substantial proportion of patients lack suitable donor vessels. Synthetic grafts perform adequately in large-diameter applications but frequently fail in small-diameter settings (≤6 mm) due to thrombosis, infection, and poor long-term patency. These limitations highlight the need for next-generation arterial grafts and robust preclinical evaluation systems.

Here, we present two complementary large-animal in vivo sheep models for systematic assessment of arterial graft safety, functionality and regenerative potential. One model enables implantation of short interposition grafts (≈10 cm) in the carotid artery, while a second, novel configuration accommodates long-segment grafts (≈30 cm) in a carotid-axillary setup. Both models are integrated with a comprehensive analytical framework to evaluate patency, regeneration, biomechanical stability, immune activation and transcriptomic remodeling.

Method overview:

• Sheep models supporting implantation of short and long arterial grafts under physiological flow

• Multimodal evaluation combining imaging, biomechanics, histology, flow cytometry, and transcriptomics

• Generic platform for assessing graft functionality, safety and biological characterization

## Graphical abstract

**Figure:**
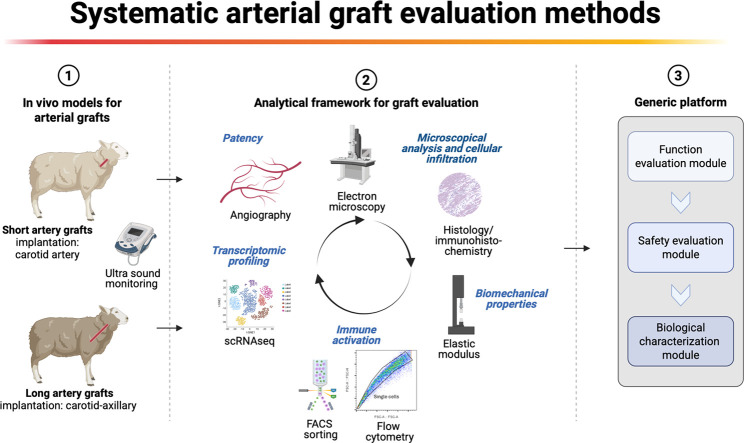

## Specifications table

**Table:** 

**Subject area**	Biochemistry, Genetics and Molecular Biology
**More specific subject area**	Pre-clinical molecular biology
**Name of your method**	Evaluation of vascular grafts
**Name and reference of original method**	Lachmi Jenndahl, Klas Österberg, Yalda Bogestål, Robin Simsa, Tobias Gustafsson-Hedberg, Patrik Stenlund, Sarunas Petronis, Annika Krona, Per Fogelstrand, Raimund Strehl, Joakim Håkansson*. Personalized tissue-engineered arteries as vascular graft transplants: A safety study in sheep. Regenerative Therapy 2022 Sep 7;21:331-341. doi: 10.1016/j.reth.2022.08.005
**Resource availability**	All information is described in the method details section.

## Background

Cardiovascular disease remains the leading cause of mortality worldwide, and although minimally invasive interventions have advanced considerably, open surgical reconstruction using vascular grafts is still required for many patients. Synthetic prostheses such as PTFE and Dacron perform well in large-diameter reconstructions [1], whereas autologous vessels remain the preferred option for small-diameter bypass procedures due to superior biocompatibility and patency. However, a substantial proportion of patients lack suitable autologous conduits. In up to one-third of patients with lower limb arterial disease, the saphenous vein is unavailable or unsuitable due to anatomical variation, prior harvesting, or poor vessel quality [2]. Harvesting procedures also prolong surgery and may cause complications such as wound infection or nerve injury [3].

Synthetic grafts show limited performance in small-diameter applications (≤6 mm), where thrombosis, occlusion and infection remain major challenges [[4], [5], [6], [7]]. Alternative biomaterials, including bacterial cellulose, have not demonstrated sufficient long-term suitability [8]. Furthermore, biomechanical mismatch between graft and native artery may induce intimal hyperplasia and graft failure, while foreign materials may trigger inflammatory or immune responses that impair integration. Tissue-engineered vascular grafts (TEVGs) have therefore emerged as a promising strategy, aiming to combine mechanical integrity with biological functionality and regenerative potential [9]. Although decellularized grafts reduce immunogenicity, complications such as aneurysm formation and thrombosis have been reported [10,11]. In contrast, individualized grafts designed to regenerate in vivo have shown encouraging preclinical results [[12], [13], [14]].

For biological medicinal products, including advanced therapy medicinal products (ATMPs), robust evaluation of safety, functionality and potency is essential [15]. Potency testing frequently requires complementary methods capable of capturing complex biological effects. Animal-based in vivo assays are recognized by ICH Q6B as valid tools for demonstrating biological activity [15]. Multiple animal models for vascular conduit evaluation exist [16], yet no single model fulfills all criteria for comprehensive assessment [17]. Large animal models are generally preferred due to their closer anatomical and physiological similarity to humans compared with small rodents [18]. While non-human primates offer high translational relevance, ethical and practical constraints limit their use [19,20]. Sheep are particularly suitable for small-diameter vascular graft assessment because their coagulation system more closely resembles that of humans than that of pigs or dogs [16,18,19]. Despite the widespread use of sheep for vascular graft evaluation, most reported models are based on carotid interposition grafting and are primarily designed for assessing relatively short graft segments. While suitable for evaluating surgical handling, patency and biocompatibility, these models provide limited opportunities to investigate long-segment arterial grafts intended for more complex vascular reconstructions. Furthermore, many studies focus on individual outcome measures, whereas standardized methodologies integrating functional, biomechanical, histological, immunological, and molecular characterization remain scarce.

To address these limitations, we present two complementary sheep models for arterial graft assessment. The first model enables implantation of short grafts up to approximately 10 cm [13,21], in the carotid artery and serves as a robust platform for assessing graft safety and functionality under physiological arterial flow conditions. The second model introduces a novel axillary-carotid configuration that accommodates grafts of up to approximately 30 cm, thereby expanding the range of vascular substitutes that can be evaluated in vivo. Combined with a structured analytical workflow encompassing imaging, biomechanics, histology, immune profiling, flow cytometry and transcriptomic analyses, the models provide a standardized platform for comprehensive preclinical assessment of graft performance and regenerative potential.

## Method details

### Large animal in vivo models

All animal procedures performed to illustrate the models and methods were made in accordance with the Guidelines for Care and Use of Laboratory Animals of Sweden and Spain according to the Directive 2010/63/EU of the European Parliament and have been approve by the local ethics committee for animal studies at the administrative court of appeals in Gothenburg, Sweden and Caceres, Spain. The models described in this manuscript have been applied and refined through several independent preclinical studies. The surgical procedures and analytical workflows are therefore well established and have demonstrated robust reproducibility.

Here, we present two in vivo models for evaluating arterial grafts: one designed for shorter grafts of up to approximately 10 cm, and a novel model developed for longer grafts of up to approximately 30 cm. The anesthesia protocol is the same for both models. The studies were performed in adult female sheep (>2 years of age) of the Merino and Texel breeds, with body weights ranging from 44 to 77 kg at the time of surgery. Animals were housed at accredited large-animal research facilities where the studies were conducted. Environmental conditions, including temperature, humidity, and ventilation, were maintained in accordance with national regulations and institutional animal welfare guidelines. The animals were maintained on a 12 h light/12 h dark cycle and had access to both indoor housing and outdoor paddocks within the animal facility. Sheep were group-housed whenever compatible with the study design, fed a standard commercial ruminant diet, and provided free access to water throughout the study period.

Anticoagulation: Clopidogrel (Plavix, Sanofi) 75 mg oral administration and Dalteparin (Fragmin, Pfizer) 12,500 IU for animals ≤ 65 kg or 15,000 IU for animals ≥ 65 kg, subcutaneous administration is initiated 48h before surgery. Clopidogrel is administered once daily throughout the study period, while Dalteparin is administered once daily during the first postoperative month unless otherwise dictated by study-specific requirements. The treatment is administered in the morning, except on the day of surgery, when Dalteparin is given in the afternoon or evening .

Perioperative anticoagulation: Immediately before arterial clamping, unfractionated heparin (Leo Pharma, 5,000 IU, intravenous administration) is administered.

Anesthesia: Premedication consisted of an injection of Dexmedetomidine (15 µg/kg, Dexdomitor, Orion Pharma Animal Health). Anesthesia is induced with (20–40 mL, 10 mg/mL, Promea Vet, Orion Pharma Animal Health) and maintained using inhaled isoflurane (Attane Vet, VM Pharma) delivered through endotracheal intubation.

Analgesia: Buprenorphine (30 µg/kg, Vetergesic Vet, Orion Pharma Animal Health) and Carprofen (4 mg/kg, Norocarp Vet, N-Vet) are administered intramuscularly preoperatively and continued postoperatively according to veterinary assessment

Antibiotic prophylaxis: Amoxicillin (15 mg/kg, Vetrimoxin Vet, Cava Animal Health) is administered before skin incision via intramuscular injection and repeated according to veterinary assessment.

The surgical procedures should be performed under sterile conditions.

**Fig. 1:**
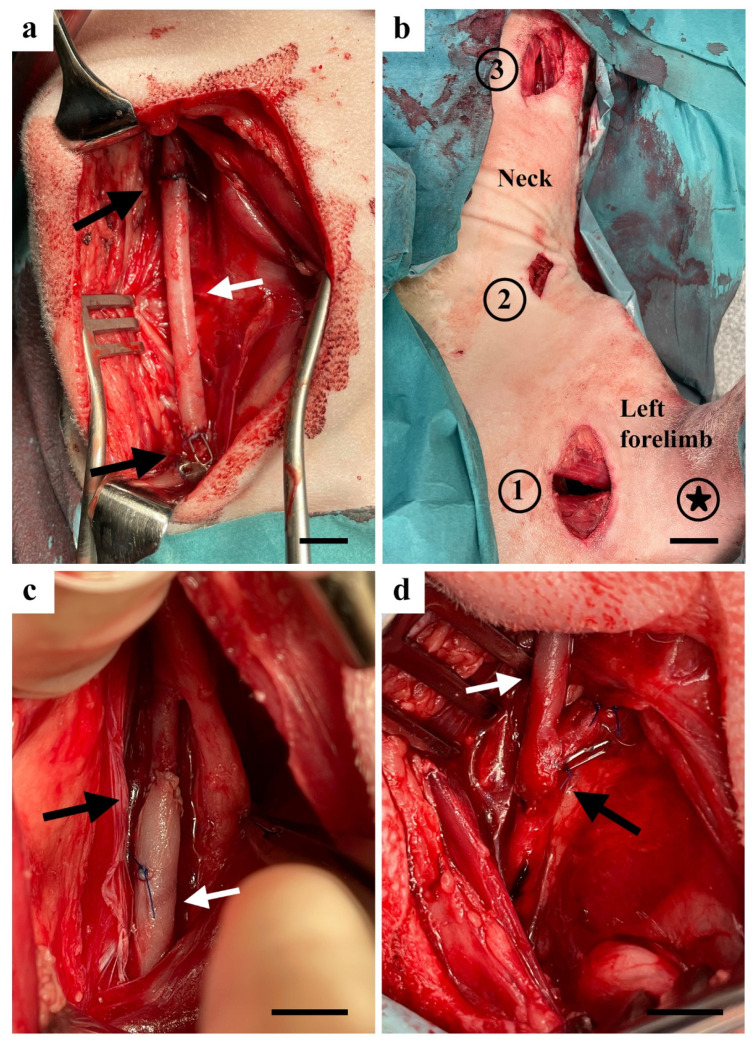
Surgical insertion of graft in sheep models. a) End-to-end anastomosis (black arrows) of arterial graft (white arrow) up to approximately 10 cm on carotis artery. b-d) End-to-side anastomosis of arterial graft up to approximately 30 cm from the axillary to the carotid artery. b) Cranial direction upwards, and caudal direction downwards in the image. The star indicates the front left limb. 1 = surgical site at the axillary artery, 2 = middle surgical site in the cervicothoracic junction region to simplify tunneling between carotis and the axillary artery, 3 = surgical site at the carotid artery. c) Close up image from site 1 in (b) where the graft (white arrow) is anastomosed (black arrow) to the carotid artery using a continuous suture. A metal clip is sutured outside the anastomosis for orientation during x-ray. d) Close up image from site 3 in (b) where the graft (white arrow) is anastomosed (black arrow) to the axillary artery using a continuous suture. A metal clip is sutured outside the anastomosis for orientation during x-ray. The native artery is ligated caudally to the anastomosis. Scale bars are 1 cm in a, c and d, and 5 cm in b.

Surgery protocol for arteries up to approximately 10 cm: An incision is made on the left side of trachea and arteria carotis is dissected free from surrounding tissue. At the time of graft implantation, heparin 5000 IU (Leo Pharma) is administered intravenously before clamping of the artery. Up to approximately a ten cm segment of graft material is implanted by end-to-end anastomosis to the native carotid using 6-0 Prolene (Ethicon) single sutures. Importantly, depending on the mechanical properties of the graft material, the grafts should be stretched when anastomosed to the native artery to prevent the graft from folding, which could lead to occlusion. To accomplish this, a short segment of the carotid artery can be excised to assure the graft is correctly placed. This segment should be about a 50% length of the graft at most. As a surgery control, sham operated carotid artery can be used. In the sham-operation, the procedure is performed in the same way, but after dissected free, the native artery is cut twice at a distance of up to 10 cm and then sutured back. Metal clips can be sutured outside of the blood vessel at the anastomosis sites for facilitated orientation under x-ray imaging, Fig. 1a. The soft tissue is closed with 3-0 Vicryl (Ethicon) and the skin is closed with a 3-0 Monocryl (Ethicon) suture intradermally.

Surgery protocol for arteries up to approximately 30 cm: The left thoracic limb is extended cranially and abducted. A physical depression is palpated on the caudal side of the transverse pectoral muscle in the axilla, and a 10 cm incision is made in a craniocaudal direction. Dissection continues through loose connective tissue in a plane between the latissimus dorsi and deep pectoralis muscles, leading into the axillary space, where the axillary artery is identified by palpation. The artery is carefully dissected and isolated from the surrounding tissue. An incision is made on the left side of trachea, and the carotid artery is dissected free from surrounding tissue. An additional incision is made between the axillar and carotid incisions and tunneling is performed dorsal of the deep pectoral muscle with fingers in combination with forceps to continue subcutaneously to the carotid artery, Fig. 1 b. Prior to clamping the artery, 5000 IU of heparin (Leo Pharma) is administered intravenously. A graft segment, up to approximately 30 cm in length, can be implanted in this model. One end of the graft is anastomosed end-to-side to the axillary artery using a continuous suture with 7-0 Prolene (Ethicon). The graft is then clamped, and the axillary arterial clamps are released to restore blood flow to the forelimb. The graft is controlled for rotation and then sutured to the carotid artery through an end-to-side anastomosis using a continuous suture with 7-0 Prolene (Ethicon). The carotid artery is ligated proximal to the anastomosis before the vascular clamps are removed and the perfusion is restored through the axillary-carotid bypass. Metal clips can be sutured outside of the blood vessel at the anastomosis sites for facilitated orientation under x-ray imaging, Fig. 1 c and d. The soft tissue is closed with 3-0 Vicryl (Ethicon) sutures, and the skin is sutured intradermally with 3-0 Monocryl (Ethicon).

In this methods description, we demonstrate the use of arterial donor material for vascular grafting in the in vivo models, and we propose that the model could also be applied to a wide range of other graft materials such as superficial and deep veins, synthetic grafts and bio-engineered grafts. The platform is not limited to arterial donor tissue and may be applied to a broad range of vascular graft materials, including autologous and allogeneic arteries, superficial and deep veins, decellularized biological grafts, tissue-engineered vascular grafts, synthetic vascular prostheses (e.g., ePTFE and Dacron), and hybrid bioengineered constructs. The primary requirement is that the graft possesses sufficient dimensions and mechanical integrity to permit surgical implantation and withstand physiological arterial blood flow and pressure during the study period.

Peripheral blood samples are preferably collected from the jugular vein, and this can be done at different time points while the sheep is awake.

### Ultrasound and angiography

**Fig. 2:**
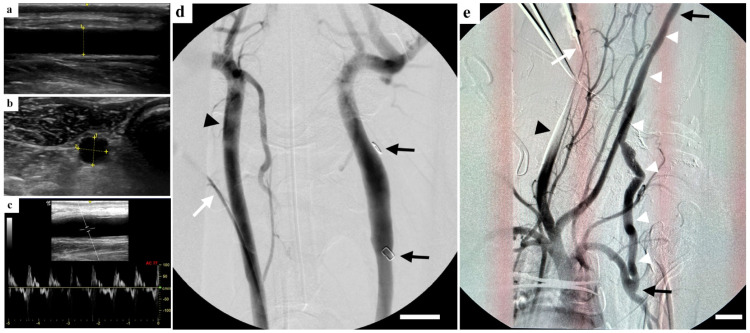
Ultrasound and angiography analysis to assess luminal diameter and graft patency. a and b) Ultrasound (B-mode) assessment longitudinally (a) and as cross-section (b), respectively. c) Blood flow velocity assessment P-wave with ultrasound. d and e) Angiography of the sheep neck region in the short d) and long e) arterial graft model. In d, e), the native contralateral artery that did not have surgery is indicated by a black arrowhead. In d), the carotid artery with the interpositioned graft between the black arrows indicating the anastomoses. The metal clips sutured outside the anastomoses for orientation during angiography are visible. In e), the graft (marked by the white arrowheads) is placed between the axillary and the carotid artery. The black arrows indicate the anastomoses. The white arrow in d, e) indicates the cannula through which contrast fluid is injected. Scalebars are 2 cm.

After transplantation, graft patency and functionality can be monitored longitudinally using ultrasound and angiography. Ultrasound enables repeated, non-invasive assessments while the sheep is awake and can be performed immediately after surgery (to obtain a baseline measurement) and at pre-defined follow-up intervals throughout the study. Suggested intervals are one week, one month and then every three months post-surgery. In addition to evaluating graft patency, ultrasound allows repeated measurements of the luminal diameter both longitudinally and in cross-section, as well as assessment of vessel morphology and blood flow characteristics, Fig. 2 a) and b). Here, we used Ultrasound (General Electric, LOGIQ e R8) in color doppler mode to identify blood vessels and B-mode to define vessel wall anatomy and for diameter measurements. Pulse wave (PW) mode is used to confirm arterial blood flow by detecting bi-phasic pulse wave velocity and peak velocity between 50-100 m/s, Fig. 2 c). Angiography is preferably performed before euthanasia but can also be conducted at different time points during the study, requiring the sheep to be under anesthesia. Here, digital subtraction angiograms were performed with C-arm fluoroscopy system. The administration of contrast fluid can be achieved through two alternative approaches. 1) The contralateral carotid artery is cannulated, and contrast fluid (Urografin 76, Bayer) is injected into the aortic arch enabling visualization of both the native carotid artery and the vascular graft on the angiogram (Fig. 2d and e). 2) An alternative for visualization of the long graft - the axillary artery can be accessed endovascularly via a femoral artery approach, navigating through the abdominal aorta, thoracic aorta, and brachiocephalic trunk under fluoroscopic guidance.

### Scanning electron microscopy

**Fig. 3:**
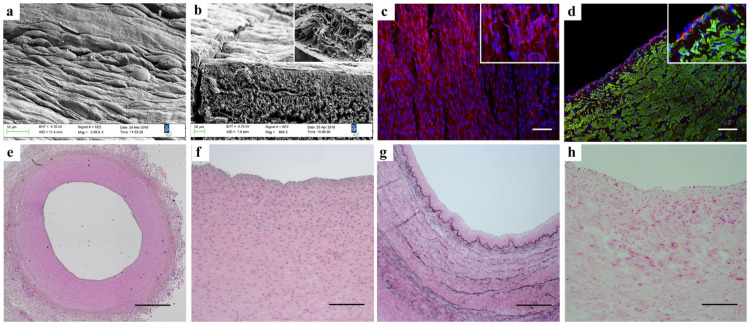
Examples of Scanning electron microscopy (SEM), immunofluorescence and histological characterization of vascular graft. a, b) SEM images of (a) endothelial cells on the luminal surface and (b) on the blood vessel wall cross-section visualizing the extracellular matrix structure. c, d) Immunohistochemical stainings with antibodies against CD31 for visualization of endothelial cells (red), alpha smooth muscle actin for smooth muscle cells (green) and DAPI for cell nuclei (blue). c) is *en face* of the luminal surface and d) cross-section of the blood vessel wall. Insets are higher magnification. e, f) Hematoxylin and eosin staining of cross-section of blood vessel for illustration of cellular density and morphology. g) Verhoeff Van Gieson showing elastin elastic fibers in black and h) Von Kossa staining of blood vessel cross-section. Mineralization will appear as black, but in this section from a native carotid artery there is no mineralization. Insets in b–d represents higher magnification. Scale bars are 10µm in a, 20µm in b, 50µm in c and d; 100 µm in e, f; 200 µm in g and h.

Scanning electron microscopy (SEM) imaging of fixed blood vessels is performed using, for example, a Zeiss Supra 40VP instrument in secondary electron imaging mode, 4–5 keV electron acceleration, 8–12 mm working distance, and ×100-×5000 magnification. The fixation of the samples (2–5 mm long ring segments of blood vessel, sliced across to reveal inner surface) is carried out in 2.5% glutaraldehyde (Sigma-Aldrich), followed by 0.5% osmium tetroxide (Sigma-Aldrich) for at least 24 hours. The fixed samples are then rinsed in Milli-Q water, dehydrated in ethanol series (30 %-100 %), rinsed in hexamethyldisilazane (Thermo Fischer Scientific), and dried in a flow hood for 48 h. Prior to imaging, the samples are mounted on microscopy pins using conductive adhesive pads and coated by 15 nm Au/Pd metallic film using Gatan PECS Model 682 sputter-coater to prevent charging when exposed to electron beam, Fig. 3 a and b).

### Histology and immunohistochemistry

After euthanization of the animal, the operated arterial segment containing the graft and native carotid artery segments (as control), are excised for further histological and biochemical analysis. Samples with cells are fixed in formaldehyde (Histofix, Histolab) for 24–72 hours, embedded in paraffin, and sectioned at a thickness of 5 μm. Immunofluorescence staining is performed on paraffin sections by rehydrating the samples and retrieving antigens through incubation in a Tris-EDTA buffer (10 mM Tris Base, 1 mM EDTA, 0.05% Tween 20, pH 9.0) in a 95°C water bath for 15 minutes. The sections are blocked with 10% fetal bovine serum (FBS, Gibco) at room temperature for 30 minutes and then incubated overnight at 4°C in a humidified chamber with primary antibodies. A variety of antibodies targeting different proteins can be used, among which CD31 (1:25, mouse, ab28364, Abcam) is the most commonly used marker for endothelial cells, and alpha smooth muscle actin (αSMA, 1:100, rabbit, ab7817, Abcam) the most used marker for smooth muscle cells and pericytes. Following incubation, the sections are washed three times for 5 minutes in PBS and subsequently incubated for 1 hour with secondary antibodies e.g. conjugated to Alexa Fluor 488 or 594 (anti-mouse: 1:200, A21202, ThermoFisher; anti-rabbit: 1:200, A21207, ThermoFisher). Slides are then mounted with ProLong Antifade mounting medium (ThermoFisher, USA) and visualized using a fluorescence microscope, Fig. 3 c and d). For general morphology, sections are rehydrated using standard protocols and stained with 25 μg/mL DAPI (4′,6-diamidino-2-phenylindole) or hematoxylin-eosin (H&E) to identify nuclei within the tissue, Fig. 3e and f). In addition to H&E staining, suitable complementary stainings are Verhoeff-van Gieson to visualize elastin fibers, Fig. 3g), and von Kossa to detect potential calcium deposits in the graft., Fig. 3 h).

### Biomechanics analysis

**Fig. 4:**
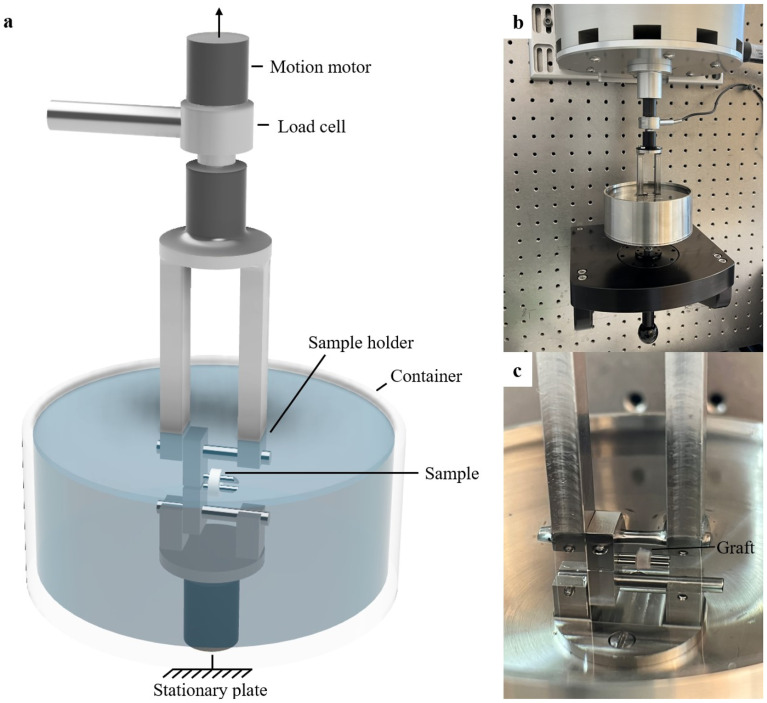
Example of setup for biomechanical evaluation. a) A schematic drawing of the biomechanical setup for the ring tensile test, including the motion motor connected to the load cell, the pin sample holder, and the PBS container attached to the lower stationary plate. b) The complete actual tensile test setup. c) A magnification of the pin holder submerged in PBS with a ring segment mounted, awaiting testing.

The complex structure and composition of the vascular wall varies with the type of vessel and introduces intricate non-linear viscoelastic biomechanical properties. Over the years, a large variation in mechanical characterization has emerged that differs in test type, protocol and data analysis making comparison between studies difficult. Selecting appropriate tests and parameters to characterize these materials can be challenging, but it has been recommended to use tensile tests to compare failure mechanics early on during the development and thereafter move on to mechanical responses at physiological conditions [22]. Herein, the focus is on the material properties of arteries and arterial grafts, specifically burst pressure and the circumferential Young's incremental elastic modulus (stiffness at local circumferential stresses and strains) [23]. These properties are determined using a ring tensile test to compare the graft materials to native tissue and verify that the grafts can withstand the internal pressure. Each artery is divided into subsections, and the one selected for biomechanical analysis is rested for at least 2 hours to allow for tissue contractions to loosen before being tested. The tissue is cut into ring segments, 3 mm in length, and measured using a digital caliper. Replicate samples of at least 3 are advised. Ring tensile testing is conducted in PBS solution at room temperature using a tensile tester, Fig. 4 a) and b) (e.g., the Planar Biaxial TestBench Instrument. TA Instruments ElectroForce System Group). One at a time, the rings are mounted on two metal pins, one stationary and one moving at a constant speed, Fig. 4 c). Samples are preloaded to 0.03 N, adapted for 30 seconds, and then strained until failure at 40 mm/min while unrestricted in the axial direction.

**Fig. 5:**
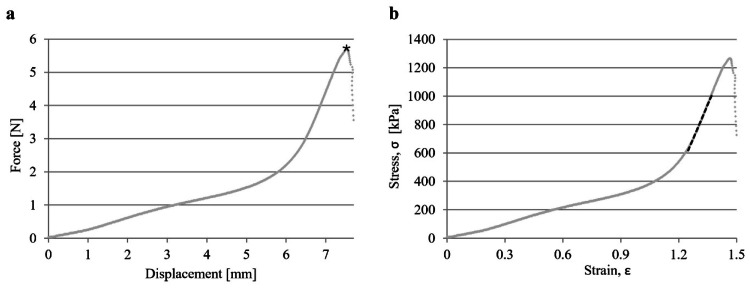
Examples of force-displacement and stress-strain plots obtained during biomechanical assessment of native carotid arterial tissue. a) A force-displacement plot illustrating the burst pressure estimation point indicated by an asterisk, here corresponding to approximately 1700 mmHg. b) A stress-strain plot illustrating the local slope at 50–80% of the maximum force, indicated by a dotted line, used to estimate the circumferential incremental elastic modulus (EInc), here approximately 3.1 MPa.

Load and displacement are recorded, and the burst pressure estimated by Laplace's law which has been shown to conform with a direct pressurization test when calculated using the force and internal diameter at failure [24]. Burst pressure, failure circumferential stress and strain, and circumferential incremental elastic modulus (EInc) are calculated from the data, with EInc estimated for high loads (50–80% of maximum force) using the sample unloaded cross-sectional area, see Fig. 5 a) and b). The contralateral animal model enables pair-wise statistical comparison between untreated (native) samples and graft samples within the same animal minimizing variation between animals. Other factors that may influence the results and add to the variation are reproduction of ringlets, heterogeneity in ringlet size and wall thickness which can also be challenging to estimate. The fact that blood vessel tissue is strain rate dependent needs to be accounted for if e.g., the mechanical properties at physiological pressures are studied. Test types, protocols and analysis methods need to be synchronized before comparison of results between studies is appropriate [22]. Recommendations for further comparison include direct pressurization testing at physiological conditions.

### Dissociation of cells

A protocol for dissociating vein tissue is detailed in [14]; we present here a modified version adapted for arterial tissue. To rinse residual blood from the tissue, the artery is washed several times with HBSS dissociation solution (HBSS-DS, Thermo Fisher Scientific). 1.5-2 cm of artery is minced into 2-3 mm pieces with a sterile scalpel or scissors in a 6-well plate. 2 ml digestion buffer, containing 200 U/ml Collagenase I (Thermo Fisher Scientific), 100 U/ml Collagenase II (Thermo Fisher Scientific) and 1.6 U/ml Dispase I (Sigma Aldrige) in HBSS, is added to the minced artery and incubated at 37°C for 1h in a stirring incubator (100 rpm). A nylon mech Cell strainer; 70 µm (Fisher Scientific) is placed on a 50 ml falcon tube and wetted with 2 ml of DMEM (Thermo Fisher Scientific) + 20% FBS (Thermo Fisher Scientific). Make sure that the whole surface area of the strainer is wet. The digested tissue is transferred to the nylon mesh with the use of serological pipettes (2 ml). The strainer is rinsed with an additional 1 ml DMEM+20% FBS, followed by rinsing the 6-well with 2 ml DMEM+FBS that is transferred to the nylon mesh. The cells are transferred to a 15 ml tube. The 50 ml tube is rinsed with an additional 2 ml DMEM+FBS and added to the 15 ml tube. The cell suspension is centrifuged for 5 minutes at 300 x g. The supernatant is removed, and the cell pellet resuspend in 4 ml 1X Red Blood cell lysis solution (Miltenyi) for 2 minutes at room temperature. The lysis is stopped by adding 5 ml DMEM+20% FBS, and centrifuged for 5 minutes at 300 x g. The supernatant is removed, and the cell pellet resuspended in 0.5 ml FACS buffer (2% FCS in PBS). The cells are counted, and the viable cell density is determined using a Bürcher chamber and trypan blue (dil.1:2).

For total RNA sequencing, the dissociated cells are centrifuged for 5 minutes at 300 x g, the supernatant is removed, 300 µl RNAprotect Cell reagent (Qiagen) is added to the pellet and the cells are transferred to nunc tubes (Thermo Fischer Scientific) and stored at -80°C until RNA extraction.

For single cell sequencing, the dissociated cells are fixated using the Evercode Cell Fixation kit (Parse Bioscience) according to the manufacturer’s instructions. Briefly, a minimum of 50,000 cells are transferred into a 5 ml polypropylene eppendorf tube, followed by centrifugation for 10 minutes at 300 x g at 4°C. The supernatant is discarded, and the cell pellet resuspend in 187.5 ul cold Cell Prefixation Buffer (prepared according to manufacturers) or Cell Prefixation Buffer + BSA. The cells are pipetted through a 40 μm strainer (Fisher Scientific) into a new 5 ml polypropylene centrifuge tube and stored on ice. 62.5μl of Cell Fixation Solution is added to the 5 mL tube and mixed immediately by pipetting up and down 3 times with a 1000µl pipette set to 250 μl. The cell suspension is immediately incubated in refrigerator for 10 minutes. 20 μl of Cell Permeabilization Solution is added to the 5 mL tube, mixed by pipetting up and down 3 times with a 1000 µl pipette set to 250 μl and incubated in refrigerator for 3 minutes. 1 mL of Cell Neutralization Buffer is added to the 5 mL tube and inverted once to mix, and centrifuged for 10 minutes at 300 x g at 4°C. The supernatant is discarded, and the pellet resuspend in 37.5μl of cold Cell Buffer. 1μl of DMSO is added and the tube gently flicked 3 times to mix, before incubation in refrigerator for 1 minute. Once again, 1μl of DMSO is added and the tube gently flicked the tube 3 times to mix, before incubation in refrigerator for 1 minute. The cell suspension is mixed by pipetting up and down 5 times with a 200 µl pipette set to 75 μl (avoid creating bubbles). It is critical to not vortex the cells. The cells are counted, transferred to nunc frees tubes and stored at -80°C. During the first 24 hours, the tubes should be placed in freezing containers (e.g., Mr. Frosty Nalgene or CoolCell) to ensure a gradual and controlled freezing process.

### Sorting of endothelial cells with the use of FACS

To isolate endothelial cells from fibroblasts, leukocytes, muscle or blood cells in the dissociated cells, Fluorescence-activated cell sorting (FACS) is used together with antibodies against CD31 and CD45. CD31, also known as PECAM-1 (platelet endothelial cell adhesion molecule-1), is one of the most commonly used endothelial cell markers; however, certain leukocytes also express CD31. Thus, to separate endothelial cells from leukocytes, CD45, also known as leukocyte common antigen, is used and endothelial cells are sorted out as CD31+CD45- cells. After counting, the dissociated cells are centrifuged for 5 minutes at 300 x g and the supernatant is removed. The cell pellet is resuspended in 200µl FACS buffer, directly stained with anti-sheep CD31 PE (BioRad) and anti-sheep CD45 (BioRad), and incubated for 30 minutes in refrigerator. Following incubation, the cells are centrifuged for 5 minutes at 300 x g, the supernatant is removed and 2ml FACS buffer is added to wash the cells. Once again, the cells are centrifuged at 300 x g for 5 minutes at room temperature, the supernatant is removed and FACS buffer is added for an additional wash step. After the second centrifugation, cells are stained for 5 minutes at room temperature with Red Nucleic Acid Stain (BD Bioscience) diluted 1:8×106 in 500µl FACS buffer.

**Fig. 6:**
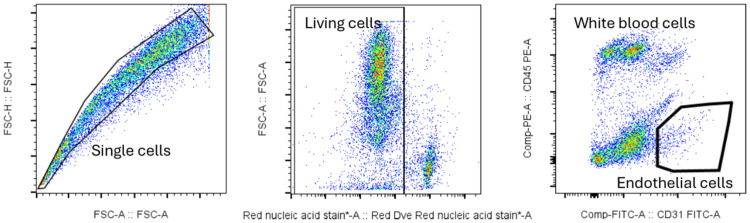
Gating strategy for sorting arterial endothelial cells. Dissociated cells from the artery are stained with anti-CD31 FITC, anti-CD45 PE antibodies and red nucleic acid stain to identify and sort living endothelial cells that express CD31 but not CD45. First, to exclude cells that are adhering to each other, single cells are identified with the use of FSC-H vs FSC-A (left FACS plot). The single cells are thereafter gaited for living cells that are identified as negative for red nucleic acid stain (middle column). The living endothelial cells are identified as CD31+CD45- in the right FACS plot.

The cells are finally single-cell sorted according to the gating strategy shown in Fig. 6, on for example a BD FACS Melody using the ACDU system (Automated Cell Deposition Unit) into 96 well plates containing CelluLyser Micro Lysis buffer (TATAA, #H104) for further analysis.

### RNA preparation

For total cell sequencing, RNA is prepared using the RNeasy micro kit (Qiagen) according to the manufacturer’s instructions. Briefly, the cells are centrifuged for 5 minutes at 5,000 x g and lysed using buffer RLT with added β-Mercaptoethanol. An on-column DNase removal step is added and the RNA eluted in 14 µl RNase free water. RNA concentration and quality are established using Qubit 4 (Thermo Fisher) and Nanodrop 2000 (Thermo Fisher). For library preparation the RNA concentration should be 1 ng-1 μg and the A260/280 ratio should be between 1.8 and 2.1. The samples are stored at -80°C until library preparation.

### Next generation sequencing

#### Total cell sequencing

For total cell sequencing, 10 µl per extracted RNA sample is used for library preparation. Prior to library preparation, poly(A) mRNA is generated, the Poly(A) Selection Kit (Lexogen) is used according to the manufacturer’s instructions. Briefly, the total RNA is denatured at 60°C for 1 min and hybridized to magnetic beads selective for RNA with a poly(A) tail. UDI (Unique dual index) library preparation is performed using the Corall total RNA-seq Library prep kit (Lexogen) according to the manufacturer’s instructions. The libraries are generated through reverse transcription into cDNA and amplification and indexing are made with PCR. The indexing PCR is performed at the cycle number determined using the PCR Add-on Kit for Illumina (Lexogen) and libraries indexed using the Lexogen UDI 12nt index set (Lexogen). The libraries are eluted in 20 µl Elution buffer and stored at -20°C. Size distribution of the libraries is analyzed using Bioanalyzer 2100 HS DNA kit (Agilent) according to manufacturer’s instructions. Libraries are diluted 1:10000 and quantified with qPCR on a CFX96 (BioRad) using the KAPA Library Quantification Kit – Illumina (Roche) according to the manufacturer’s instructions. Libraries are diluted to 650pM, pooled and sequenced on a NextSeq2000 sequencer (Illumina). A 5% PhiX spike-in (Illumina) is used as control.

#### Single cell sequencing

Libraries for single cell sequencing are generated using Evercode WT (Whole Transcriptome) v3 kit (Parse Bioscience) according to the manufacturer’s instructions. Briefly, fixed and permeabilized cells are barcoded in three subsequent rounds and cDNA generated in the first round of barcoding. Sublibraries are created and indexed with Illumina Truseq 8nt UDI set and stored at -20°C. Size distribution of the sublibraries is analyzed using Bioanalyzer 2100 HS DNA kit (Agilent) according to manufacturer’s instructions. The sublibraries are quantified using the Qubit dsDNA HS (High Sensitivity) kit (Thermo Fisher) diluted to 650pM, pooled and sequenced on a NextSeq2000 sequencer (Illumina). A 5% PhiX spike-in (Illumina) is used as control.

Bulk RNA-Seq data processing and analysis workflow

**Fig. 7:**
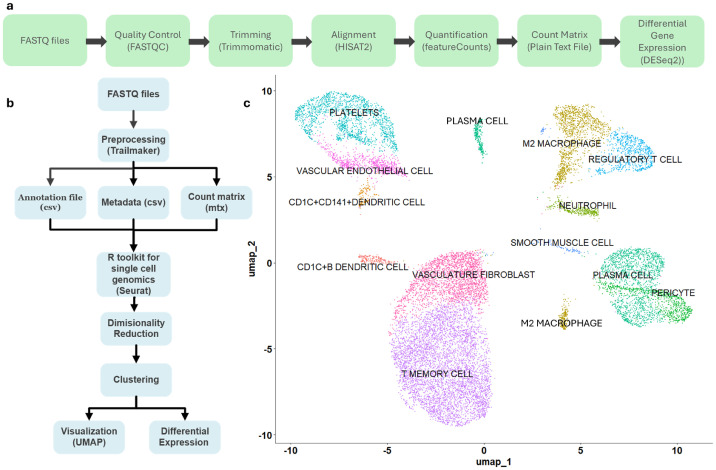
Overview of transcriptomics workflows and UMAP visualization of arterial tissue a) An overview of the RNA-seq data processing and analysis workflow. b) An overview of the data processing and analysis of the single-cell RNA-seq datasets. c) UMAP plot illustrates the distribution of cell types identified in arterial tissue.

Fig. 7a provides an overview of the RNA-seq data processing and analysis workflow described below.

#### Quality control and preprocessing

Raw RNA-seq data in FASTQ format, generated using a paired-end sequencing platform, is processed in a Linux shell environment. Initial quality control is performed using FastQC (v0.11.9) to assess base quality, sequence duplication, GC content, and adapter contamination [25]. Identified quality issues such as adapter sequences, biased 5′-end content, and low-quality trailing bases are corrected using Trimmomatic (v0.39) in paired-end mode [26]. The trimming configuration includes adapter removal, cropping the first three bases from the 5′ end, trimming low-quality bases from the 3′ end (Phred <10), and discarding reads shorter than 25 bp. Processing is parallelized across 14 threads, and both paired and unpaired reads are retained.

### Reference indexing and read alignment

The Ovis aries reference genome (Oar_v3.1, Ensembl release 112) and gene annotation file can be downloaded from the Ensembl FTP server [27]. Reference genome indices are built using the hisat2-build function from HISAT2 (v2.2.1) [28]. Trimmed reads are then aligned to the indexed reference genome using HISAT2 with 16 threads. Alignment outputs are streamed directly into Samtools (v1.14) for sorting and BAM conversion, avoiding intermediate SAM file generation [29].

### Gene quantification

Gene-level read quantification is performed using the featureCounts function from the Subread package (v2.0.3) in paired-end and unstranded mode [30]. Reads mapping ambiguously to multiple features or genomic locations are excluded. Summary statistics are used to assess the distribution of unassigned reads, including those unmapped or lacking overlap with annotated features.

### Differential gene expression analysis

#### Filtering and normalization

The raw count matrix and associated sample metadata are imported into R 4.4.0 [31] and analyzed using the DESeq2 package [32]. Genes with fewer than 10 counts in over 80% of samples are removed to reduce background noise. Normalization is performed using DESeq2’s median-of-ratios method via the estimateSizeFactors function to adjust for library size and RNA composition.

#### Model fitting and statistical testing

A DESeqDataSet object is created using a design formula based on experimental conditions. Differential expression analysis is conducted using the DESeq function, which fits a negative binomial generalized linear model with dispersion estimation and shrinkage of log2 fold changes (8). Statistical significance is determined using Wald tests. Genes with an adjusted p-value < 0.05 and biologically meaningful log2 fold changes are considered differentially expressed.

#### Annotation and interpretation

Gene identifiers are annotated using the Ensembl BioMart web interface [33]. Ensembl gene IDs are mapped to gene symbols, with preference given to UniProtKB names when available. Final count matrices, metadata, and differential expression results are prepared for visualization and functional interpretation.

#### Group comparison

To evaluate the transcriptional effects of surgical manipulation, gene expression profiles of native artery samples are compared with sham-operated controls (surgery without graft implantation). The comparison did not reveal significant transcriptomic changes related to arterial function or structural integrity. Only two genes, PTPRB and CCDC158, were found to be significantly differentially expressed (adjusted p-value < 0.05); however, neither gene is known to be involved in arterial function or vascular remodeling. These findings indicate that the sham procedure do not produce measurable effects on arterial gene expression, supporting its use as a valid control for subsequent analyses involving graft implantation.

#### Single-cell RNA-Seq data processing and analysis workflow

Fig. 7b shows an overview of the data processing and analysis of the single-cell RNA-seq datasets. The eight sublibraries containing the FASTQ sequences of the distinct sample together with the sample loading table are uploaded to the Trailmaker a web service offered by PARSE Biosciences to preprocess and analyzed single cell RNA seq generated by the Parse technology [34]. The reference genome can be selected from the dropdown menus Oar_v3.1:Ovis aries (Sheep). The pipeline is run to produce reports for the individual samples containing barcode rank plot, QC metrics including the estimated number of cell sequences and the plate heatmaps displaying transcripts and cells per well. The pipeline output includes several key data products to support downstream analysis. Count matrices are available under both the “Unfiltered matrices” and “Filtered matrices” sections. Unfiltered matrices retain barcodes with at least 10 transcripts and apply minimal filtering, making them suitable for flexible analysis workflows and serving as the default input for the Trailmaker Insights module. Filtered matrices are further refined based on thresholds determined from the barcode-rank plot in the Pipeline module. As the same thresholding is applied within Trailmaker Insights, use of the filtered matrices within that environment is not recommended. Additional outputs include Combined reports, available as a downloadable ZIP archive (all_summaries.zip), which contains HTML reports, quality control metrics (CSV), and pipeline log files. Sublibrary reports provide the same set of outputs, broken down by individual sublibrary. For advanced users, the All files option includes the complete pipeline output, including alignment BAM files, and is intended for command-line access. The platform also provides quality control metrics and basic visualizations such as Uniform Manifold Approximation and Projection (UMAP) and clustering summaries.

#### Downstream analysis and cell type annotations

The output unfiltered matrices from Trailmaker were imported into Seurat in R for downstream analysis and interpretation [35]. Cells were filtered based on quality control thresholds, including the number of detected genes, total Unique Molecular Identifier (UMI) counts, and mitochondrial gene content, to remove low-quality cells and multiplets. The data were normalized and scaled using NormalizeData default, followed by dimensionality reduction via Principal Component Analysis (PCA). The optimal number of dimensions for UMAP visualization was determined using an elbow ElbowPlot function, which guided the selection of principal components (PCs) based on the point of inflection in variance explained. Clustering was performed using the Louvain algorithm, and UMAP was used to visualize the transcriptional landscape, Fig. 7c. To generate a comprehensive database of cell type-specific marker genes, we integrated three publicly available resources: PanglaoDB [36], CellMarker 2.0 [37], and ScTypeDB_full from the ScType package [38]. PanglaoDB and CellMarker 2.0 provide manually and computationally curated marker gene lists across a wide range of human and mouse tissues, with annotations linking genes to cell types and tissues based on single-cell transcriptomic data and literature support. ScTypeDB_full, retrieved via the ScType R package, offers a high-confidence cell type-gene mapping table optimized for automatic cell type annotation, supporting both tissue-level and fine-grained immunological hierarchies [38]. For each resource, cell type–gene associations were harmonized by:

1. Standardizing cell type nomenclature using ontology references.
2. Converting gene identifiers to official HGNC gene symbols for human data.
3. Removing duplicate and ambiguous entries across datasets.
4. Merging cell type–gene pairs across sources, retaining source provenance for each marker to enable confidence weighting or prioritization in downstream analyses.

**Fig. 8:**
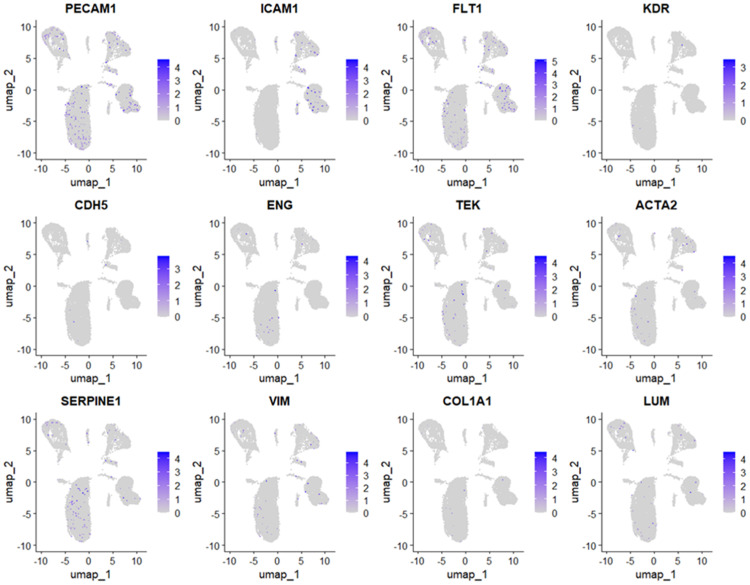
UMAP visualization of arterial tissue showing expression of cell type-specific markers. Marker genes were plotted using FeaturePlot in Seurat to assess distribution across identified clusters. Endothelial markers: PECAM1, ICAM1, FLT1, KDR, CDH5, ENG, and TEK. Smooth muscle cell markers: ACTA2, and SERPINE1. Fibroblast markers: COL1A1, and LUM. General mesenchymal marker: VIM. Expression levels are color-coded, with darker colour indicating higher expression.

Gene orthologs between human and mouse reference marker genes and their corresponding sheep genes was resolved using Ensembl BioMart [27]. The resulting integrated marker reference supports downstream annotation of clusters in single-cell RNA-seq data. The final database was saved as a .xlsx format to ensure compatibility with ScType tool. Cell types that were classified as "unknown" by the ScType tool were further analyzed using the Enrichr web-based platform to assist with cell type identification [39]. Furthermore, biomarkers for different cell types within arterial tissue were visualized using FeaturePlot (11). Endothelial cell markers included PECAM1, ICAM1, FLT1, KDR, CDH5, ENG, and TEK. Fibroblast markers included COL1A1, and LUM. Smooth muscle cell markers included ACTA2, and SERPINE1. In addition, VIM was included as a general mesenchymal marker to assess stromal cell presence, Fig. 8 [14].

#### Preparation of peripheral blood mononuclear cells

**Fig. 9:**
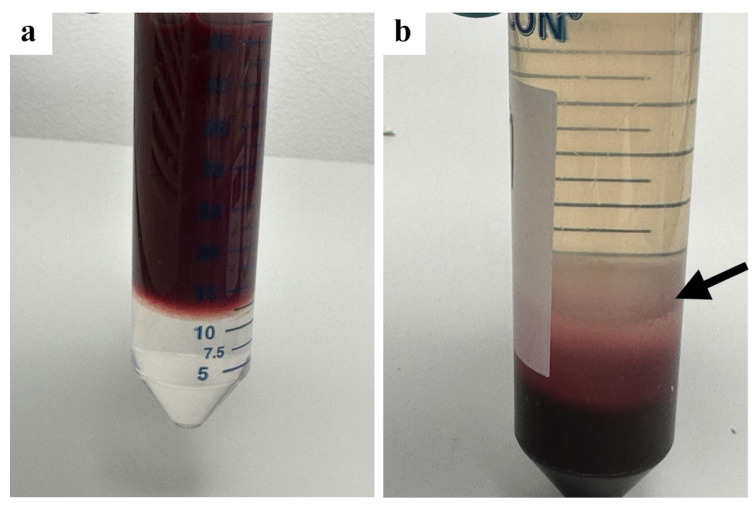
Preparation of peripheral blood mononuclear cells (PBMC) from whole blood with ficoll. a) carefully layering the blood on top of the ficoll. b) After centrifugation, the PBMC is separated as a white layer, indicated by the black arrow.

To determine if the recipient’s immune system is activated after graft implant, peripheral blood is taken from the sheep before surgery and at different time points after surgery, i.e. 1 and 2 weeks, 1-, 6- and 12-months post-surgery. Peripheral blood, e.g. from the jugular vein, is collected in EDTA tubes (Swemed) and stored at room temperature until preparation of peripheral blood mononuclear cells (PBMC), which is performed within 48 hours after blood collection. The blood is diluted in PBS 1:1 and the solution is carefully layered over the Ficoll (Ficoll-Paque, Cytiva) in a ratio 2:1, Fig. 9a. The layered blood solution/Ficoll is centrifugated in a swing out rotor at x 900 g at room temperature for 25 minutes with low to no acceleration and no deceleration. The PBMC layer, white cell layer on top of the Ficoll-section, Fig. 9b, is transferred to a new tube, washed with PBS and centrifugated at x 350 g at room temperature for 5 minutes. At this and the following centrifugation, full acceleration and deceleration can be used. The supernatant is discarded, and the pellet washed by resuspending the cells in 1 ml of PBS and then adding PBS to a final volume of 12 ml. The centrifugation step is repeated, the supernatant discarded, and the pellet resuspended in 2 ml of freezing medium (10% heat-inactivated FCS or FBS (Thermo Fisher Scientific) and 5% DMSO (Sigma-Aldrich) in RPMI cell medium (Thermo Fisher Scientific). The cell suspension is transferred to cryotubes and stored at -80°C. During the first 24 hours, the tubes should be placed in freezing containers (e.g., Mr. Frosty Nalgene or CoolCell) to ensure a gradual and controlled freezing process. After 24-48 hours the PBMC can be moved to a –150°C freezer.

#### Flow cytometry analysis

**Fig. 10:**
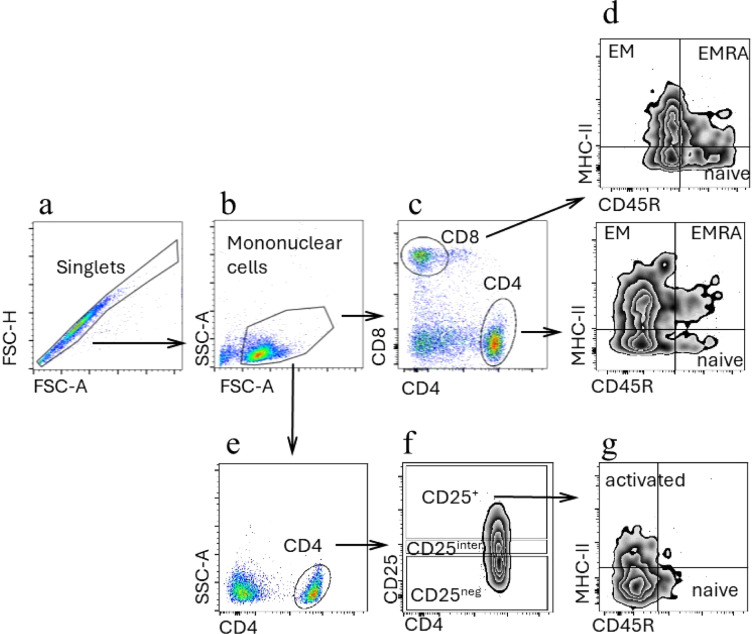
Gating strategy for circulating T cell populations. PBMC are stained with monoclonal antibodies toward CD4, CD8, MHC-II, CD45R and CD25. (a, b) Singlet PBMC cells are gated for lymphocytes. To analyze the naïve, effector cells (EM) or EM CD45R+ effector cells (EMRA) within the CD4+ or CD8+ T cells, lymphocytes are gated for either CD4 or CD8 (c) and thereafter according to their expression of MHC-II and CD45R (d). (e-f) Regulatory T cells are identified by gating lymphocytes for CD4 (e), followed by detection of CD25neg, CD25intermediate and CD25+-cell populations within the CD4+ T cells (f). The CD25+ cells are thereafter gaited for their expression of CD45R and MCH-II to detect naïve or activated Tregs, respectively (g).

The protocol for flow cytometry analysis was followed as described in Sehic et al, [40]. For cell surface staining of PMBC for T cell phenotyping, PBMC is thawed in a water bath at 37°C, and the cells are transferred carefully to a 15ml tube containing 10ml FACS buffer. The cells are centrifuged for 5 minutes at 300 x g and the supernatant is removed. The centrifugation step is repeated, and the cell pellet is stained with primary antibodies anti-sheep CD45R (Table 1) for 20 min at 4°C in dark. The cells are washed twice, by adding 2ml of FACS buffer followed by centrifugation for 5 minutes at 300 x g removing the supernatant and repeating the centrifugation above. The cell pellet is stained with a secondary anti-IgG1 antibody conjugated with BV421 (Table 1) for 20 min in the dark at 4°C. The staining is followed by a wash as described above, and finally the cells are stained with anti-sheep monoclonal antibodies (CD25, MHC2, CD4, CD45R, and CD8; Table 1) for 20 minutes in 4°C in dark. After incubation, the cells are washed twice and resuspended in FACS buffer before analysis by flow cytometry with use of BD FACS Lyric (BD Bioscience). Fig. 10 shows the gating strategy for analysis of naïve or activated T cells and Tregs in the circulating mononuclear cells from sheep blood. In our case, all data is analyzed with FLOWJO FlowJO software (Tree Star, Ashland, OR). We employ the fluorochrome minus one technique, wherein the samples are stained with all antibodies used in the assay, encompassing all fluorochromes except for one.

**Table 1:** Monoclonal antibodies used for flow cytometry analysis of different T cell populations in circulating sheep blood.

Panel to detect naïve and activated T cells			
Antibody	fluorochrome	clone	supplier
anti-CD4	AF647	OX-35	BioRad
anti-CD8	PE	38.65	BioRad
anti-MHC-II	FITC	28.1	BioRad
anti-CD45R	NA	20.96	BioRad
anti-IgG^a^	BV421	A85-1	BD Bioscience

**Panel to detect regulatory T cells**			

**Antibody**	**fluorochrome**	**clone**	**supplier**

anti-CD4	AF647	OX-35	BioRad
Anti-CD25	FITC	9.14	BioRad
anti-MHC-II	PE	28.1	BioRad
anti-CD45R	NA	20.96	BioRad
anti-IgG^a^	BV421	A85-1	BD Bioscience

a secondary antibody

## Method validation

Included in method details.

## Limitations

Not applicable.

## Ethics statements

All animal procedures performed to illustrate the models and methods were complied with the ARRIVE guidelines and made in accordance with the Guidelines for Care and Use of Laboratory Animals of Sweden and Spain according to the Directive 2010/63/EU of the European Parliament for animal experiments and have been approved by the local ethics committee for animal studies at the administrative court of appeals in Gothenburg, Sweden and Caceres, Spain. The animals used to illustrate the results in this study were all females; however, the models and methods presented are independent of sex and can be used on both males and females.

## CRediT author statement

K.Ö., Å. L., V. K. K., N. G., J. S., P. S., Y. B., S. P., S. S., V. C., F.M.S.M., C.B.D., H. R., and J. H. contributed to the study design. K.Ö., Å. L., V. K. K., N. G., J. S., P. S., Y. B., S. P., S. S., V. C., F.M.S.M., C.B.D., H. R., J. H. performed experiments and analyzed results. All authors contributed to data production and interpretation.

K.Ö., Å. L., N. G., J. S., P. S., Y. B., S. P., V. C., H. R., and J. H. wrote the initial draft of the manuscript; all authors reviewed and revised the manuscript.

## Acknowledgments

Animal experimental studies were performed in Sweden at the department of experimental biomedicine, Gothenburg University and in Spain at the ICTS ‘NANBIOSIS’ (units 10, 14, 21, 22 and 24) of the Fundación Centro de Cirugía de Mínima Invasión Jesús Usón.

This work was supported by Swedish innovation agency Vinnova project CAMP (contract no. 2017-02130), a common call by VINNOVA and Vetenskapsrådet: Biologcal pharmaseuticals (Dnr 2017-02983), and by University of Skövde under grants from the Swedish Knowledge Foundation (#2016-0330, #2020-0014).

Graphical Abstract Created in BioRender. Synnergren, J. (2026) https://BioRender.com/ls3w50g

## Declaration of interests

The authors declare that they have no known competing financial interests or personal relationships that could have appeared to influence the work reported in this paper.

## Data Availability

Data will be made available on request.
